# “Oh, You’ve Come to Visit the Yard?”: Phenotypic Capital, Intragroup Marginalization, and the Gated Sanctuary in Black LGBTQ+ Communities

**DOI:** 10.3390/bs16020292

**Published:** 2026-02-18

**Authors:** Keith J. Watts, Shawndaya S. Thrasher, Nicole Campbell, Laneshia R. Conner, Julian K. Glover, Janet K. Otachi, DeKeitra Griffin

**Affiliations:** 1 College of Social Work, University of Kentucky, Lexington, KY 40508, USA; laneshia.conner@uky.edu; 2 Center for Health, Engagement, and Transformation, College of Medicine, University of Kentucky, Lexington, KY 40536, USA; 3 School of Social Work, Louisiana State University, Baton Rouge, LA 70802, USA; sthrasher@lsu.edu (S.S.T.); dgrif36@lsu.edu (D.G.); 4 Darden College of Education & Professional Studies, Old Dominion University, Norfolk, VA 23529, USA; ncampbel@odu.edu; 5 Department of Gender, Sexuality, and Women’s Studies, Virginia Commonwealth University, Richmond, VA 23284, USA; gloverj2@vcu.edu; 6 Kent School of Social Work and Family Science, University of Louisville, Louisville, KY 40292, USA; janet.otachi@louisville.edu

**Keywords:** Black LGBTQ+, intragroup marginalization, phenotypic capital, intersectionality, community belonging, minority stress

## Abstract

Identity-based communities that share common characteristics, beliefs, and experiences (e.g., Black LGBTQ+ communities) have historically been conceptualized as protective bubbles that buffer Black LGBTQ+ individuals against the deleterious effects of systemic racism and cisheterosexism. However, this monolithic narrative often masks the internal power dynamics that divide belonging. This study explores the exclusionary dynamics embedded within these safe spaces, examining how internal hierarchies of skin tone, socioeconomic status, and gender performance function as proximal stressors. Guided by a critical constructivist paradigm, this study utilized Reflexive Thematic Analysis to analyze open-ended survey responses from 74 Black LGBTQ+ adults. Data were drawn from a larger mixed-methods study and analyzed using a six-phase recursive process to identify latent patterns of intragroup gatekeeping. The analysis revealed that the sanctuary of the community is restricted. Three primary themes emerged: (1) Phenotypic Capital and the Politics of Authenticity, where lighter skin tone triggered authenticity scrutiny and darker skin tone faced rejection based on physical appearance; (2) Socioeconomic Gatekeeping, where belonging was stratified by the cost of participation and protective insularity within working-class spaces; and (3) Policing the Binary, where rigid adherence to gender archetypes created a landscape of performance surveillance. Access to community resilience is not a universal right but a negotiated status contingent upon the payment of a resilience tax. To promote genuine health equity, researchers and practitioners working with this population must move beyond the uncritical referral to “community” and actively dismantle the internalized systems of oppression that fracture collective survival.

## 1. Introduction

The human condition is fundamentally defined by a necessity for belongingness, a pervasive, powerful, and persistent motivation to establish and maintain significant interpersonal attachments (Baumeister & Leary, 1995; Stillman et al., 2009). When individuals achieve a sense of secure social connectedness, they experience higher levels of life satisfaction; conversely, the frustration of this need (often characterized as “thwarted belongingness”) serves as a primary proximal predictor of suicidal desire, structural isolation, and a deep loss of life meaning (Joiner, 2005; Ringer & Anestis, 2018; Stillman et al., 2009). For Black sexual and gender minorities, this search for meaning is perpetually situated within a society that systematically employs erasure and systemic exclusion, rendering identity-based community connection an essential survival strategy (Jenkins, 2023; Wade & Harper, 2020).

Historically, research has conceptualized Black LGBTQ+ community spaces as sanctuaries or “protective bubbles” that buffer external social stressors, including systemic anti-Black racism and cisheterosexism (Meyer, 2003; Wong et al., 2014). This narrative suggests that shared marginalized identity provides equal access to resilience resources for all group members. However, this monolithic framework effaces the internal power dynamics of marginalized collectives (Bowleg, 2012). Navigating this complexity requires moving beyond additive models that view race plus gender as a cumulative outcome (Crenshaw, 1989) toward a reflexive intersectionality that interrogates how power relations structure the category of community (Cole, 2009; Collins, 2015). Belonging is never a static state but a status actively made through differentiation; thus, the cohesion of the group often necessitates the creation of internal “others” (Dalal, 2009).

Guided by Cohen’s (1999) theory of secondary marginalization, this study posits that marginalized groups under intense external pressure frequently enforce internal conformity by marginalizing their most vulnerable members—such as those with non-binary gender expressions or lower socioeconomic status—to maintain a respectable image for the dominant society (Cohen, 1999, 2005). This phenomenon translates into psychological distress through the mechanism of intragroup marginalization, a form of minority stress where interpersonal distancing functions as a distinct stressor (Castillo et al., 2007). When an individual transgresses the group, whether through being “too queer,” or failing to perform gender correctly, the group responds with social alienation (Castillo et al., 2007; Tajfel, 1974). Consequently, the boundaries of “Blackness” and the legitimacy of “queerness” are perpetually negotiated through a multi-axis hierarchy of phenotypic capital, socioeconomic attainment, and performative authenticity (Ellis & Destine, 2023; M. L. Hunter, 2002; Monk, 2021; Nicolazzo, 2016). This stratification is further compounded by neoliberalism, transforming community spaces into commodified sites where ‘belonging’ shifts from an inherent birthright into a transactional status reserved for those who possess the requisite capital (Brown, 2015).

### 1.1. The Psychology of Belonging: Identity Centrality and Conflict

While intragroup marginalization delineates the sociological boundaries of the community, the acute internal impact of this exclusion is best explicated through the lens of Social Identity Theory (Tajfel, 1974). For Black LGBTQ+ individuals navigating a landscape of systemic erasure, the community functions as more than a social network; it is a fundamental scaffold of the self-concept (Tropp & Wright, 2001). This cognitive and emotional overlap creates a powerful sense of linked fate (Moore, 2010), wherein the individual’s sense of safety in the world is perceived as inextricably bound to the collective. This study posits that because the racial–sexual community serves as the primary shield against the distal stressors of racism and heterosexism, rejection from this group is not processed merely as a social snub, but as a severe disruption of one’s only haven.

However, this reliance on the community creates a distinct psychological vulnerability described by the Intragroup Status and Health (ISAH) model (Begeny & Huo, 2017). The ISAH model reveals that while high identity centrality (e.g., strongly identifying with the group) is necessary to buffer against external oppression, it simultaneously renders the individual hyper-sensitive to internal rejection. The more central the community is to one’s survival, the more damaging the gatekeeping becomes. When this ostensible safe haven is revealed to be a site of status anxiety and competition, the buffering effect collapses (Pachankis et al., 2020). This forces the individual into a state of conflict in allegiances (Sarno et al., 2015), where they must psychically fragment themselves—suppressing their gender expression to satisfy racial norms, or masking their socioeconomic reality to satisfy class norms. We argue that the chronic cognitive labor of perpetual self-monitoring required to maintain eligibility for protection constitutes the psychological core of the resilience tax.

### 1.2. The Material and Phenotypic Landscape

Finally, the landscape of belonging is structured by the material realities of neoliberal capitalism and the historical legacy of colorism. The transition to neoliberalism has accelerated the fragmentation of LGBTQ+ identities, consolidating a respectable middle-class gay identity while marginalizing working-class and impoverished members (Drucker, 2011). Within this paradigm, outness is revealed not as a universal developmental stage but as a resource-dependent strategy dictated by social class (Taylor, 2016). Those with professional and educational capital are afforded a life perceived as socially legitimate, and the safety to be visible, while working-class and impoverished members face a classing of their queer lives that renders them invisible or improper (McDermott, 2011; Seidman, 2011).

Simultaneously, colorism remains a pervasive force that stratifies the Black experience from within (Monk, 2021). Grounded in the historical legacies of European colonialism and slavery, skin tone functions as color capital—a form of social currency that systematically privileges lighter-skinned individuals (Ellis & Destine, 2023; M. L. Hunter, 2002). Empirical research consistently demonstrates a light-skin wage premium and better health outcomes, while darker-skinned individuals face significantly higher rates of reported discrimination and social exclusion (Goldsmith et al., 2007; Monk, 2015). Within Black LGBTQ+ social spheres, this hierarchy is often intensified through internalized colorism and the digital amplification of racialized sexual discrimination (Wade & Harper, 2020), where Black bodies are either fetishized or explicitly excluded based on their proximity to Whiteness (Brennan et al., 2013; Hall, 2018; Wade & Harper, 2020). Thus, the community functions as a marketplace where phenotypic and economic capital dictate the terms of admission.

### 1.3. The Current Study

Despite the burgeoning literature on intersectionality, a significant gap remains regarding the ways internal community hierarchies function as proximal stressors for Black LGBTQ+ adults (Monk, 2021). While previous work established a quantitative link between community belongingness and mental health (Watts & Thrasher, 2023), it did not unpack the granular, interactional processes that constitute exclusion. This study fills this gap by utilizing a Reflexive Thematic Analysis (RTA) framework to interrogate the open-ended survey responses of 74 Black sexual and gender minority adults. Consistent with the exploratory nature of Reflexive Thematic Analysis, this study did not test a priori hypotheses but rather sought to generate new conceptual understandings of community dynamics. The analysis was guided by the following research questions: (1) How do Black LGBTQ+ adults describe their experiences of belonging and exclusion within their racial-sexual communities? (2) What latent patterns of intragroup dynamics emerge from participants’ narratives regarding access to these spaces? and (3) In what ways do these lived experiences align with or complicate the prevailing narrative of the community as a protective bubble?

## 2. Materials and Methods

### 2.1. Study Design and Data Collection

The data for this analysis were drawn from a larger, mixed-methods study conducted in 2021 at a large, public research university in the Mid-Atlantic United States (Watts & Thrasher, 2023). The original study was designed to explore the broad relationship between community belongingness and mental health among Black LGBTQ+ adults. Data collection utilized a cross-sectional, web-based survey design hosted on REDCap, which allowed for the recruitment of a geographically diverse sample often unreachable through traditional venue-based sampling (Meyer & Wilson, 2009). Recruitment was restricted to individuals who met the following formal eligibility criteria: (1) self-identifying as Black or African American; (2) self-identifying as a sexual or gender minority (LGBTQ); (3) being 18 years of age or older; and (4) currently residing in the United States. Recruitment strategies were purposive and multi-pronged, leveraging digital networks including listservs of Black LGBTQ+ organizations, social media platforms, and professional networks.

The qualitative data for the current analysis consists of responses to five open-ended questions selected from the larger survey to specifically explore community experiences. While open-ended survey responses typically yield less volume than semi-structured interviews, they often encourage a crystallization of experience where participants distill complex phenomena into their most salient components. Given the sensitive nature of intragroup marginalization, this anonymous format likely reduced social desirability bias, allowing participants to candidly name painful internal community dynamics (e.g., colorism) that might be softened in a face-to-face interview. Specifically, participants were asked: (1) “Describe your first experience of LGBTQ community”; (2) “Describe your first experience of Black LGBTQ community”; (3) “Was it challenging or difficult to physically access these spaces? If so, why?”; (4) “How have your feelings of belongingness to a Black, LGBTQ, and/or Black LGBTQ community supported your ability to cope with social stressors you have experienced because of these identities?”; and (5) “How have your feelings of belongingness to the Black, LGBTQ, and/or Black LGBTQ community affected your overall well-being?” The study protocol received university Institutional Review Board approval and is available (Watts & Thrasher, 2023).

### 2.2. Study Participants

The analytic sample consisted of 74 Black LGBTQ+ adults who provided rich, narrative responses to open-ended survey items regarding their experiences of community access and belonging. Participants ranged in age from 18 to 64, with a mean age of 31.3 years (*SD* = 8.09), capturing perspectives from both emerging adults navigating identity formation and older adults negotiating aging in the margins (Chen et al., 2022). Geographically, the sample captured a diverse cross-section of the United States, moving beyond a single regional context. While a plurality of participants resided in the Mid-Atlantic region (e.g., Virginia, Maryland, Washington D.C.; n = 34), the sample included significant representation from the Southern (e.g., Georgia, Texas, Tennessee; n = 16), Midwestern (e.g., Illinois, Michigan, Ohio; n = 11), and Western (e.g., California, Arizona; n = 7) regions. This broad geographic distribution reflects the efficacy of the digital recruitment strategy in reaching Black LGBTQ+ adults outside of traditional urban coastal enclaves. Despite this regional diversity, the sample was predominantly metropolitan, spanning the urban-rural continuum with a heavy skew toward urban (55.4%) and suburban (31.1%) centers, while rural participants (12.2%) remained underrepresented. Gender identity was similarly heterogeneous, including cisgender men (n = 30), cisgender women (n = 23), and a significant cohort of transgender and gender expansive individuals (n = 21). The sample size of 74 provided sufficient information power for the analysis, as the participants’ narratives were characterized by high communicative quality and were directly relevant to the study’s specific theoretical aims (Malterud et al., 2016).

### 2.3. Epistemological Stance

This study is grounded in a critical constructivist paradigm, which posits that reality is socially constructed through historical and cultural dialogues, yet constrained by material structures of power (Lincoln & Guba, 1985). Unlike post-positivist approaches that seek to minimize researcher influence to achieve objectivity, this inquiry embraces a “Big Q” qualitative orientation (Braun & Clarke, 2021a). Here, the researcher’s subjectivity is not a contaminant but a vital analytic resource—a theoretical lens shaped by professional expertise in social work and lived experience within Black LGBTQ+ communities (Berger, 2015). The study employs an RTA design to move beyond the semantic surface of participant responses and interrogate the latent, underlying assumptions that govern intragroup belonging (Braun & Clarke, 2006, 2021b).

### 2.4. Data Analysis: The Six-Phase Reflexive Approach

Data analysis followed the six-phase recursive process outlined by Braun and Clarke (2006, 2021b):

1. Familiarization: We began by immersing ourselves in the data through repeated, active reading of the raw qualitative exports. This phase involved noting initial analytic observations and distancing the qualitative responses from their quantitative context to appreciate each narrative on its own terms. The first author kept a reflexive journal during this stage to bracket his own assumptions as an insider-researcher.
2. Systematic Coding: We employed a collaborative, reflexive coding strategy consistent with Big Q qualitative inquiry. Rather than seeking inter-rater reliability or enforcing consensus, practices which Braun and Clarke (2021b) identify as incoherent with the interpretive nature of RTA, we utilized multiple coders to facilitate collaborative reflexivity. The first and second authors coded the data independently to generate initial impressions, then met to discuss where their interpretations diverged. These divergences were not treated as errors to be resolved but as analytic resources that revealed the complexity of the data. For example, where one author interpreted a participant’s withdrawal as “avoidance”, the other interpreted it as “protective boundary setting”. Through reflexive dialogue, we integrated these perspectives to develop richer, more nuanced codes (e.g., “protective insularity”) that captured the multifaceted nature of the participant experience.
3. Generating Themes: In this phase, we collated the generated codes into potential themes, moving from descriptive labels to interpretative constructs. This involved examining how codes combined to form overarching patterns of meaning. For instance, codes related to “judgment at the bar”, “skin tone scrutiny”, and “authenticity testing” were clustered together to form the candidate theme of “Phenotypic Capital”. We visualized these relationships using thematic maps to ensure that the candidate themes represented a coherent story about the data.
4. Reviewing Themes: We then engaged in a two-level review of the candidate themes. First, we checked if the themes worked in relation to the coded extracts (Level 1), ensuring that each theme was supported by sufficient data. Second, we reviewed the themes against the entire dataset (Level 2) to ensure they accurately reflected the meanings present in the dataset as a whole. This process led to the refinement of the “Resilience Tax” concept, ensuring it was grounded in participant descriptions of exhaustion and labor rather than just theoretical inference.
5. Defining and Naming Themes: Each theme was then refined to identify its essence and scope. We developed clear definitions and names for each theme to ensure theoretical clarity and distinct boundaries. For example, the theme initially labeled “Gender Rules” was refined to “Policing the Binary: The Enforcement of Gender Roles” to more accurately capture the active, surveillance-based nature of the gatekeeping described by participants.
6. Producing the Report: Finally, we wove the analytic narrative together with vivid data extracts to produce a coherent argument that answers the research questions. This phase involved selecting the most compelling quotes to illustrate the themes and relating the analysis back to the existing literature on intersectionality and minority stress, demonstrating how the findings extend current theoretical understanding.

### 2.5. Positionality and Reflexivity

Adhering to the Big Q qualitative paradigm, the first author conducted this inquiry through the lens of the researcher-as-instrument, acknowledging that his social location served as the primary filter for data synthesis. As a Black, LGBTQ-identified scholar and professor of social work at a research institution, he occupies a unique insider-outsider status. His shared racial and sexual identity, along with cultural intuition, facilitated the decoding of latent meanings in community vernacular and the exploration of painful topics such as colorism. However, this positionality necessitated the reflexive bracketing of his class privilege; as an academic with high educational attainment, he remained hyper-aware of how his middle-class respectability might inadvertently align with the socioeconomic gatekeeping he sought to investigate. Through reflexive journaling and an active commitment to anti-oppressive praxis, he strove to ensure that the analysis did not impose a presumptuous narrative of community as refuge onto participants who experienced these spaces as sites of exclusion.

The second author identifies as a Black, cisgender woman and scholar who approaches this inquiry through a lens of dual positionality. As a Black woman, she is situated as both a racial and gender minority with lived experience navigating systems of structural oppression. While she does not identify as a sexual minority or gender-expansive—occupying an outsider status regarding the specific lived experiences of LGBTQ+ individuals—she shares the racial identity of the participants, situating her as an insider to the systemic racism and structural inequities navigating Black life. She acknowledges that her educational and professional status affords specific privileges and power within the research context. However, her lived experience within a marginalized racial community fosters a deep commitment to cultural humility, transparency, and accountability in interpreting participant narratives. As an active ally and researcher who has extensively studied Black LGBTQ+ belongingness, she utilized this unique insider-outsider perspective to critically analyze the data while remaining vigilant against imposing heteronormative biases.

### 2.6. Trustworthiness

To ensure the rigor and integrity of this inquiry, we adhered to the criteria established by Lincoln and Guba (1985). Credibility was supported through thick description of participant narratives, allowing readers to determine the resonance of the findings with the data. Transferability was addressed by providing detailed demographic and geographic context for the sample, enabling future researchers to assess the applicability of findings to other settings. Dependability was maintained via an audit trail of the analytic process, documenting the evolution of codes and themes during collaborative meetings between the first and second authors. Finally, confirmability was achieved not by eliminating researcher subjectivity, but by making it explicit through the reflexive bracketing described above. Additionally, the final reporting of these findings adheres to the Consolidated Criteria for Reporting Qualitative Research (COREQ) checklist (Tong et al., 2007).

## 3. Findings

The analysis of the qualitative data reveals a divergence from the idealized protective bubble narrative. While participants universally acknowledged the potential of the Black LGBTQ+ community to serve as a bulwark against white supremacy and cisheterosexism, their lived realities were characterized by a sophisticated system of internal regulation. Through our inductive coding process, two central conceptual patterns crystallized from the data: *phenotypic capital* and the *resilience tax*. We identified phenotypic capital as the emergent social currency associated with skin tone, where participants described both lighter and darker skins being policed against a rigid standard of racial authenticity. Furthermore, we conceptualize the resilience tax not as a metaphor, but as the cost of belonging observed in our data. Specifically, this tax appeared as: (1) chronic self-monitoring of gender performance (e.g., hiding “feminine things”); (2) the strategic concealment of class status to avoid friction; and (3) the affective depletion reported by participants who found community spaces “exhausting” or “stressful” rather than restorative.

While these concepts represent the currencies and costs of belonging, the broader structural domains of gatekeeping operated through three distinct themes: (1) Phenotypic Capital and the Politics of Authenticity, (2) Socioeconomic Gatekeeping and the Price of Admission, and (3) Policing the Binary: The Enforcement of Gender Roles. Quotes are accompanied by demographic descriptors (age, gender, sexual identity, residential geography) to situate the findings.

### 3.1. Theme 1: Phenotypic Capital and the Politics of Authenticity

The first theme provides evidence of phenotypic capital, in which skin tone functions as a form of social currency that can be converted into, or deny access to, material or social resources. It is important to note that while “color capital” in the broader society typically confers linear advantages to lighter-skinned individuals (Ellis & Destine, 2023; Goldsmith et al., 2007; M. L. Hunter, 2002; Monk, 2015), our data revealed a distinct lack of such advantage within these specific community spaces. We did not identify narratives where lighter skin was described as an asset for community entry. Instead, it consistently functioned as a friction point requiring the negotiation of authenticity. Conversely, darker skin was valued as a marker of authentic Blackness but simultaneously penalized in sexual/romantic marketplaces, illustrating the volatile, context-dependent nature of phenotypic capital.

#### 3.1.1. Sub-Theme 1a: The Fair-Skinned Burden of Proof

Lighter-skinned participants described a unique form of thwarted belongingness where their racial allegiance was perpetually interrogated. Unlike the external racism they faced in white spaces, this internal scrutiny functioned as a test of loyalty. This dynamic was vividly articulated by a participant whose narrative deconstructs the safe space myth of the Black gay bar:

“My first experience was when I went to [name of bar], the LGBT bar… [name of bar], in my opinion, almost ruined my entire outlook of the LGBT community. It was soo judgmental and I felt that I couldn’t really belong… Being a fairer skinned black person superseded my LGBT status in a black majority LGBT bar. It was clear I wasn’t welcomed… anyone who is not the status quo of African American lineage, must deal with micro aggressive comments… for instance, a light skinned person like myself must hear ‘Oh your not really black’ or ‘oh, you’ve come to visit the yard [N-word]s?’”

(age 30, Gay Man, rural)

This extract demonstrates that phenotypic capital in this context works inversely; lighter skin tone becomes a marker of the outsider. The phrase “superseded my LGBT status” suggests that within the Black LGBTQ+ enclave, racial phenotype is the primary master status, overriding the shared bond of sexual orientation. The “micro-aggressive comments” function as identity surveillance, casting the lighter-skinned individual as a tourist in their own community.

#### 3.1.2. Sub-Theme 1b: The Barrier of Complexion

For these participants, the “yard” functions as a metaphor for an exclusive racial territory where their entry is viewed as an intrusion rather than a homecoming. Historically, the term “yard [N-word]” stems from the division of labor assigned to enslaved Africans in North America, where labor was hierarchically divided between the “house” (closest to white proximity), the “yard,” and the “field”. By deploying this slur against a light-skinned individual Black person, the speaker weaponizes this historical hierarchy to mark the lighter-skinned individual as an outsider to the what is perceived as the authentic Black experience of the field/yard. Another participant reinforced that this exclusion is not merely a social slight but a fundamental barrier to access: “The challenge is being a light skin black person… sometimes your complexion. Some people are not always nice so that would be a challenge.” (age 49, Man, Gay, Suburban). Such gatekeeping creates a unique identity crisis for those whose phenotype renders them illegible to the group. As one mixed-race participant shared, this often results in a total erasure of social presence:

“I think I’m constantly in a middle state—an absence of belonging that is more ambivalent than distressing. Being a trans man who is mixed black/white, I find that there aren’t too many spaces for me. I’m rather invisible which at times is a super power and other times pretty lonely.”

(age 42. Trans Man, Queer, Urban)

These statements corroborate Monk’s (2021) findings on the ceaseless significance of skin tone, suggesting that internal hierarchies create a structural isolation where the individual is too Black for white spaces but not Black enough for the racial enclave. Gonlin (2020) identifies this as an “racial identity mismatch,” where a rigid archetype of the Black racial category allows for little fluidity, causing reflected race (i.e., how one is seen) to clash with internal self-concept, further intensifying the stress of rejection.

### 3.2. Theme 2: Socioeconomic Gatekeeping and the Price of Admission

The second theme exposes the materialist fissures in the community, revealing how belonging was often tiered based on educational attainment, professional status, and disposable income.

#### 3.2.1. Sub-Theme 2a: Protective Insularity and the Ballroom Scene

A striking finding emerged regarding the House and Ballroom community. Often romanticized in academic literature as an unconditional space of belonging, participants described a dynamic where educational privilege became a liability. As one individual shared, “The ball room scene. It’s fun but if you are educated or middle class you can feel excluded.” (age 43, Man, Gay, Suburban).

However, other participants nuanced this exclusion, framing it as a necessary survival strategy. One participant noted that this boundary maintenance is intentional: “The ball [room] was not [accessible] as the ballroom scene maintains a pretty high amount of insularity for a number of reasons including safety” (age 29, Non-Binary, Pansexual, Urban). This suggests a form of protective insularity, likely designed to protect the space from the perceived judgment of the dominant class. Participant narratives appear to describe a form of intragroup marginalization that sanctions behaviors associated with the dominant culture, such as higher education or professional speech. In this context, educational capital transforms from a societal asset into a community liability.

#### 3.2.2. Sub-Theme 2b: The Cost of Participation

Conversely, participants seeking entry into more formalized BIPOC queer organizations reported that economic precarity was a definitive wall. Belongingness was described not as an emotional state, but as a transaction:

“The best experiences that I have had have been with bipoc queer orgs. It’s just a matter of finding them and having the time/money to join their events. Also a matter of aiding in organizing events to build community.”

(age 23, Non-Binary, Bisexual; Pansexual/Queer, Urban)

This quote validates what Taylor (2016) calls the “classing of queer lives”. The requirement of time and/or money to access safe spaces highlights how neoliberalism has commodified community. For working-class Black LGBTQ+ adults, the intentionality required to find community—driving hours to a city, paying cover charges—functions as a socioeconomic gatekeeper. Furthermore, the expectation to “aid in organizing” suggests that belonging is often contingent on labor; one must produce the community to be part of it, a burden that disproportionately weighs on those with limited resources. For others, the gatekeeping was even more explicit, manifesting as a requirement for specific educational credentials. One participant noted their primary connection to the community was through a digital space restricted to “queer, black, degreed, single, millennials” (age 26, Woman, Pansexual, Rural), suggesting that “degreed” status functions as a prerequisite for social visibility.

### 3.3. Theme 3: Policing the Binary: The Enforcement of Gender Roles

The final theme illuminates the strict enforcement of gender archetypes. Despite the community’s theoretical rejection of heteronormativity, the data reveals a strict adherence to gender scripts.

#### 3.3.1. Sub-Theme 3a: Gender Archetypes

Participants described a social environment where ambiguity was not tolerated. To exist socially was to be categorized: “It appeared that clearly defined roles were important in relationships. You had to be enough or popular to exist so to say. E.g., stud or femme and various cliches.” (age 41, Woman, Same-Gender Loving, Suburban). The phrase “you had to be enough… to exist” is ontologically significant. It suggests that those who exist in the fluid spaces between “stud” and “femme”—particularly non-binary individuals—face a form of social erasure. This aligns with Nicolazzo’s (2016) findings on the “hard line to walk” for Black non-binary collegians. The community replicates the dominant society’s demand for categorization, utilizing cliches as cognitive shortcuts to determine who is a valid romantic or social partner.

#### 3.3.2. Sub-Theme 3b: Surveillance and the Gender Uniform

For those who do fit a category, the pressure to maintain that performance functions as a source of chronic intraminority stress (Pachankis et al., 2020). One participant articulated the anxiety of this constant gender surveillance: “I don’t believe in labels but I would be labeled as a stud. People always look at my clothes before my face. I feel like I’m being judged before I even open my mouth.” (age 35, Woman, Lesbian, Suburban).

Participants described how this policing often began early in their development, creating a lasting internal conflict. One participant vividly recalled projecting their own internalized anti-femininity onto a drag performance:

“I was immediately put off by the drag performance because I was projecting my fear of displaying my own femininity… This was one of the first instances where I began to understand my trauma of growing up in a predominantly black community and having to hide my desire to enjoy feminine things”.

(age 23, Man, Gay, Urban)

For others, the criteria for exclusion were multifaceted, where gender non-conformity compounded with body politics. As one participant noted of their first experience in a club setting: “I was not accepted fully due to being feminine and plus size” (age 32, Man, Gay, Urban), illustrating how the community’s gaze polices multiple axes of identity simultaneously.

This policing extends beyond those assigned female at birth. Men in the sample also reported that belonging was conditional on the performance of a specific racialized masculinity. One participant described his alienation as a result of failing to meet these fetishized standards: “[I experienced] exclusion because of my race, less desirability compared to men of other races, especially considering I did not embody the masculinity black men are fetishized for” (age 26, Man, Gay, Urban). In this context, the resilience tax is paid in the currency of gender conformity, where members must suppress their authentic expressions to remain desirable or even visible within the group.

## 4. Discussion

### 4.1. The Ontological Crisis of the Gated Community

This study was predicated on a critical interrogation of the assumption that identity-based communities function as uniform sanctuaries against structural oppression. The findings presented here detract from this reductionist narrative, offering instead qualitative empirical validation of the paradox of belonging. As hypothesized by Tate et al. (2025), our findings support the notion that, for Black LGBTQ+ adults, high levels of community investment can amplify the distress of minority stress when that community engages in exclusionary gatekeeping. While the need to belong remains a necessity for psychological survival (Baumeister & Leary, 1995), Scarpa (2025) distinguishes this from the need for significance—the feeling that one is a subject of value rather than merely an object in a setting.

Our analysis suggests that for these participants, the community isn’t always an unconditional safe haven. Instead, it often operates like a restricted marketplace where belonging must be “bought” with the right status. In this environment, access to safety is not guaranteed; it is a privilege reserved for those who hold the specific racial, economic, or gendered currency the group values. This tax is the psychological and material cost levied through the social pressure of conformity. The data indicate that the Black LGBTQ+ community is not merely a refuge but a contested site where the matrix of domination is internalized and operationalized (Collins, 2000). Collins (2000) posits that power is not just structural but operates within the domain of everyday social interaction. Our findings confirm that Black LGBTQ+ spaces function as microcosms of this domain, where members scrutinize one another using the same hierarchies of skin tone, class, and gender performance that oppress them externally. By illuminating the specific, interlocking functions of phenotypic capital, socioeconomic gatekeeping, and gender monitoring, this study argues that the community functions less as an unconditional home and more as a status-based marketplace, where belonging is an exclusive commodity good accessible primarily to those who possess the requisite racial, economic, and gendered currency.

### 4.2. Phenotypic Capital and the Politics of Racial Authenticity

The emergence of colorism as a primary determinant of belonging complicates the foundational assumption of racial solidarity. While quantitative analyses confirm that light-skinned Black men typically sit atop a three-tiered hierarchy of income and status in the broader labor market (Reece, 2021), our findings reveal a complex reversal of this capital within the Black LGBTQ+ enclave. For lighter-skinned participants, the community did not function as a respite from racial discrimination but as a site of authenticity policing. This finding validates M. Hunter’s (2007) argument that while light skin buys status in the white world, it often purchases suspicion in the Black community, where darker skin is regarded as more ethnically authentic. This suggests that phenotypic ambiguity triggers a defensive kinship logic, mirroring the internal gatekeeping described by Battle and Ashley (2008), in which the group protects its boundaries by interrogating those who physically resemble the oppressor. Conversely, the corporeal barrier experienced by darker-skinned participants, particularly in the desirability hierarchy of gay social spaces, confirms the enduring power of colorism. The fetishization and rejection described by participants align with the concept of racialized sexual discrimination, illustrating that the liberated spaces of queer nightlife and digital networking are actually sites where the Eurocentric beauty standard is most rigidly enforced (Wade & Harper, 2020).

### 4.3. Neoliberal Fracturing and the Classing of Queer Safety

Simultaneously, the fragmentation of belonging by educational and economic status expands our understanding of outness. Our data suggest that the politics of visibility is often a resource-dependent privilege. As Taylor (2016) argues, there is a “classing” of queer lives where the ability to claim a legible, celebrated LGBTQ+ identity is frequently reserved for those who possess specific forms of material and cultural capital. Furthermore, the exclusion of educated or middle-class individuals from these spaces suggests a form of protective insularity. This aligns with Drucker’s (2011) theory of neoliberal fracturing, which suggests that as a “respectable” middle-class gay identity consolidates, alternative sexual cultures may fortify their boundaries against the intrusion of normative, professionalized queer subjects.

### 4.4. Realness and Transnormativity

Finally, the enforcement of the stud/femme binary illustrates the hard line that Black gender-expansive individuals must walk (Nicolazzo, 2016). By demanding that individuals “be enough” of a stereotype to exist socially, the community replicates the cisnormative logic of the dominant society (Gibbs, 2025). This finding aligns with Nicolazzo’s (2016) conceptualization of transnormativity, which functions as an internal border that delegitimizes non-binary identities, forcing a choice between identity concealment or social expulsion. This dynamic is inextricably linked to racialized sexual objectification, where the Black trans body is scrutinized for its ability to convincingly pass as one gender or the other rather than accepted for its humanity. The findings suggest that this scrutiny may be driven by a collective anxiety about representation. Dalal (2009) frames this as the work of the “zealot”, a figure who must constantly patrol the community’s boundaries to ensure that a single line of thought persists, thereby preventing the disintegration of the group’s identity. Consequently, the freedom of the queer community is circumscribed by the same gender essentialism it claims to resist, leaving those who refuse the binary in a state of thwarted belongingness.

Despite these barriers, this study reframes such struggles not solely as deficits, but as sites where participants’ strategic agency through the calculated negotiation of identity—emphasizing their Blackness in some spaces, their queerness in others, and their status in yet others, to maximize safety and resource acquisition.

### 4.5. Implications for Practice, Policy, and Theory

For mental health clinicians, the finding that community spaces can function as sites of affective depletion necessitates a paradigm shift from standard cultural competence toward structural competency (Metzl & Hansen, 2014). Cultural competence often treats communities as benevolent, monolithic cultures to be engaged; however, our identification of a resilience tax suggests that for some clients, these spaces are sites of labor rather than refuge. Therefore, the clinical practice of uncritically referring isolated Black LGBTQ+ clients to broad identity-based supports must be re-evaluated. Given our finding that policing the binary creates chronic stress for non-binary individuals (Theme 3), clinicians should utilize intersectional eco-mapping to assess a client’s specific fit with a potential space. This involves explicitly asking: Does this client possess the phenotypic, economic, or gender capital required to be safe in this specific environment? Therapeutic goals should shift away from seeking validation from status-conscious macro-communities, which we found often demand a high cost of conformity. Instead, interventions should focus on the cultivation of micro-communities, small, chosen families where admission is based on shared values rather than the performative adherence to the racial and gender archetypes identified in this study. This approach prioritizes the quality of connection over the quantity of network ties, protecting the client’s psychological resources.

At the organizational level, leaders must actively dismantle the material and phenotypic barriers identified in Themes 1 and 2. Our finding that “degreed” status and financial costs (Theme 2) can function as gatekeepers indicates that neoliberalism has commodified belonging. To address this, organizations should restructure funding models to utilize grants and donor subsidies, thereby removing the cover charge of community participation that disenfranchises working-class members. Furthermore, the prevalence of colorist rhetoric (e.g., the “yard” slur identified in Theme 1) indicates that organizations should move beyond performative diversity statements, such as the visual inclusion of darker-skinned bodies in promotional materials without a corresponding redistribution of executive power or the codification of anti-colorist bylaws. Policy reform must include updating bylaws to explicitly name and prohibit intragroup discrimination, specifically colorism and anti-Blackness, as forms of harassment. Programmatically, this requires a deliberate redistribution of visibility. Organizations should intentionally center the leadership of darker-skinned and gender-expansive members to disrupt the desirability hierarchies that our participants described as pervasive in both physical and digital spaces.

Theoretically, this study contributes to intersectionality research by offering the resilience tax not merely as a metaphor, but as an operationalizable conceptual framework for understanding health disparities within marginalized groups. While previous work established that belonging impacts health (e.g., Watts & Thrasher, 2023), this qualitative inquiry explains how the mechanics of exclusion operate. We assert that resilience is not a free, limitless resource; rather, it is a metabolic process that consumes psychological energy. Current theoretical models often treat community connection as a universally positive variable. However, this study argues that for those on the margins of the margin, the cognitive load required to navigate specific internal gatekeepers, such as the phenotypic capital demands of colorism (Theme 1) and the gender surveillance of transnormativity (Theme 3), constitutes a chronic stressor. Future research should attempt to empirically measure this tax, calculating how the energy spent on navigating these proximal intragroup stressors depletes the reserves available for coping with external structural oppression. Ultimately, this advances the ISAH model by positioning these internal stressors not as anomalies, but as central features of the minority experience. This shift requires a perpetual, reflexive commitment to dismantling boundaries within collective lives, recognizing that the matrix of domination is not just something that happens to the community, but something that can be reproduced by it—a paradox where safety is often purchased at the cost of diversity.

### 4.6. Limitations and Future Research

While this study offers critical insights into the internal mechanics of community gatekeeping, several limitations must frame the interpretation of these findings. First, the utilization of a purposive sampling strategy leveraged through digital networks and advocacy organizations introduces specific selection biases. Our sample is likely skewed toward individuals who are already connected to LGBTQ+ networks and possess the digital literacy and technology required to complete a web-based survey. Consequently, the voices of the digitally divided, including those experiencing homelessness, older adults without online access, and individuals who are entirely disconnected from formal community structures, are likely underrepresented. Their experiences of exclusion may differ significantly from the narratives captured here. Rather than facing a gated community, these disconnected individuals may face a community desert where no such structures exist to be gated.

Second, regarding transferability, the geographic distribution of our sample presents a specific context for our findings. While the data captured a national cross-section (i.e., 18 states), there remains a plurality of participants from the Mid-Atlantic region and a heavy skew toward urban and suburban centers. Rural participants were significantly underrepresented. Therefore, the concept of the resilience tax as a cost of entry may be an urban-centric phenomenon. In rural contexts, where Black LGBTQ+ infrastructure is often non-existent, the primary stressor may be isolation rather than the status-based competition described by our participants. Furthermore, specific cultural metaphors identified in our analysis, such as “the yard”—a term linked to the Southern Black experience—may function with different intensity or meaning in regions with different racial histories, such as the Pacific Northwest or New England.

Third, while open-ended survey responses allowed for a breadth of candid, anonymous disclosure regarding sensitive intragroup topics, this method precludes the interactive depth of (semi-)structured interviewing. While we were able to identify the presence of the resilience tax, we could not probe the minute-by-minute metabolic cost of this tax in real-time conversation. Future research should employ venue-based sampling to recruit unconnected populations and utilize longitudinal or ethnographic methods to empirically measure how the cognitive load of phenotypic capital and gender surveillance depletes psychological resources over time.

## 5. Conclusions

The Black LGBTQ+ community is neither a utopia nor a monolith; it is a human institution, vulnerable to the same hegemonies of race, class, and gender that define the American experience. This study reveals that for many, the community functions less as an unconditional sanctuary and more as a status-based marketplace, where belonging is purchased through the currency of phenotypic, economic, and gendered capital. By exposing the resilience tax required to navigate these internal borders, from the historical sting of the “yard” slur to the modern exclusion of “degreed” gatekeeping, we challenge the idealized narrative of the protective bubble. However, rather than negating the necessity of community, these findings clarify the terms of its survival. To build a truly health-promoting enclave, we must move beyond the performance of inclusion to the hard work of structural competency. Only by dismantling the internalized hierarchies of skin tone, class, and gender can we transform the community from a site of conditional respite into a genuine source of mattering and validation for all its members.

## Data Availability

The data presented in this study are available on request from the corresponding author. The data are not publicly available due to privacy and ethical restrictions.

## Abbreviations

The following abbreviations are used in this manuscript:

BIPOC	Black, Indigenous, and People of Color
ISAH	Intragroup Status and Health
LGBTQ+	Lesbian, Gay, Bisexual, Transgender, Queer, and other sexual and gender identities
RTA	Reflexive Thematic Analysis
SD	Standard Deviation
